# 3D brain tumor segmentation using a two-stage optimal mass transport algorithm

**DOI:** 10.1038/s41598-021-94071-1

**Published:** 2021-08-10

**Authors:** Wen-Wei Lin, Cheng Juang, Mei-Heng Yueh, Tsung-Ming Huang, Tiexiang Li, Sheng Wang, Shing-Tung Yau

**Affiliations:** 1 Department of Applied Mathematics, National Yang Ming Chiao Tung University, Hsinchu, 300 Taiwan; 2 Electronics Department, Ming Hsin University of Science and Technology, Hsinchu, 304 Taiwan; 3 Department of Mathematics, National Taiwan Normal University, Taipei, 116 Taiwan; 4 School of Mathematics, Southeast University, Nanjing, 211189 People’s Republic of China; 5 Nanjing Center for Applied Mathematics, Nanjing, 211135 People’s Republic of China; 6 Department of Mathematics, Harvard University, Cambridge, USA

**Keywords:** Tumour heterogeneity, Applied mathematics

## Abstract

Optimal mass transport (OMT) theory, the goal of which is to move any irregular 3D object (i.e., the brain) without causing significant distortion, is used to preprocess brain tumor datasets for the first time in this paper. The first stage of a two-stage OMT (TSOMT) procedure transforms the brain into a unit solid ball. The second stage transforms the unit ball into a cube, as it is easier to apply a 3D convolutional neural network to rectangular coordinates. Small variations in the local mass-measure stretch ratio among all the brain tumor datasets confirm the robustness of the transform. Additionally, the distortion is kept at a minimum with a reasonable transport cost. The original $240 \times 240 \times 155 \times 4$ dataset is thus reduced to a cube of $128 \times 128 \times 128 \times 4$, which is a 76.6% reduction in the total number of voxels, without losing much detail. Three typical U-Nets are trained separately to predict the whole tumor (WT), tumor core (TC), and enhanced tumor (ET) from the cube. An impressive training accuracy of 0.9822 in the WT cube is achieved at 400 epochs. An inverse TSOMT method is applied to the predicted cube to obtain the brain results. The conversion loss from the TSOMT method to the inverse TSOMT method is found to be less than one percent. For training, good Dice scores (0.9781 for the WT, 0.9637 for the TC, and 0.9305 for the ET) can be obtained. Significant improvements in brain tumor detection and the segmentation accuracy are achieved. For testing, postprocessing (rotation) is added to the TSOMT, U-Net prediction, and inverse TSOMT methods for an accuracy improvement of one to two percent. It takes 200 seconds to complete the whole segmentation process on each new brain tumor dataset.

## Introduction

The introduction of the Multimodal Brain Tumor Image Segmentation Benchmark (BRATS)^1–3^ has generated enormous research interest in the image processing and machine learning (ML) community, as has the BRATS challenge, which is held regularly (for a comprehensive review, see^4^). This dataset, created by a group of distinguished neurologists, has quickly become one of the leading medical datasets due to its openness, robustness and regular maintenance. Accordingly, the automated segmentation of brain tumors, which would greatly benefit patients, has been sought by many researchers. In the early stage, top-rank performers used random forest classification^5–7^. Later, a convolutional neural network (CNN), with two layers proposed by Zikic et al.^8^ and a CNN with eight layers proposed by Randhawa et al.^9^, gained traction. More sophisticated CNN structures are being developed. Ronneberger et al.^10^ first introduced U-Net. Kamnitsas et al.^11^ presented an ensemble of multiple models and architectures, which consists of two fully convolutional networks (FCNs) and a U-Net. Isensee et al.^12^ have also shown the resiliency of the U-Net model, which is the most widely used model in the BRATS challenge.

It is expected that a 3D CNN produces a higher accuracy than its 2D counterpart because valuable information along the z-axis is taken into consideration^4,13^. However, the GPU memory constraint is the main issue^14^ given that the size of each BRATS image is $240 \times 240 \times 155 \times 4$, which represent the *x*, *y*, and *z* coordinates of the brain scan and the 4 scan modalities [fluid-attenuated inversion recovery (FLAIR), T1-weighted (T1), T1-weighted contrast-enhanced (T1CE), and T2-weighted (T2) magnetic resonance imaging (MRI)], respectively. To address the problem of memory shortage in 3D operations, Casamitjana et al.^15^ applied a 3D U-Net using data reshaped to $140 \times 140 \times 140$, which suits the memory limit. However, by reducing its dimensionality, detailed information could be lost. Isensee et al.^16^ trained their network using a randomly sampled patch size of $128 \times 128 \times 128$ with 16 filters^10^, which are expected to cover entire regions of size $240 \times 240 \times 155$. This approach has the benefit of retaining all of the information but needs more voxels than the original images. Thus, properly addressing the 3D structure is crucial for the implementation of 3D segmentation.

The optimal mass transport (OMT) theory has widely been applied in various fields, such as image processing^17–19^, data compression^20^ and generative adversarial networks^21^. The OMT problem, a pioneering work that considers minimizing the transportation cost to move a pile of solid mass from one place to another without losing any detail, was first raised by Monge in 1781 (see^22^ for details). In 1948, in light of the linear programming technique, Kantorovich^23^ relaxed the transport map to a transport scheme/plan by a joint probability distribution and first proved the existence and uniqueness of the OMT problem. Due to their excellent work in the optimal allocation of scare resources, Kantorovich and Koopmans shared the Nobel Prize in Economics in 1975. Then, in 1991, Brenier^24^ began studying the characteristics, uniqueness and existence of the OMT map and developed an alternative scheme for solving the OMT problem with a quadratic distance as the cost function for a special class of convex domains. Compared to the Monge–Kantorovich approach, the Monge-Brenier optimization method can significantly reduce the number of unknown variables from $O(n^2)$ to *O*(*n*), where *n* is the number of discretized sample points on the target domain and can solve the optimal transport map via the gradient descent method of a special convex function. For the theoretical OMT problem, in the 1990s, Caffarelli^25^ solved the regularity condition of the OMT map. Ganbo and McCann^26^ proposed the geometric interpretation of the OMT problem, and Evans^27^ expressed the OMT problem via partial differential equations (PDEs). A very nice review “Optimal Transport: Old and New” summarizing the contributions of predecessors was published by Villani^28^. For the numerical OMT problem, in 2017, Su et al.^29^ proposed a volume-preserving mesh parameterization between a simply connected 3D tetrahedral mesh $\mathcal {M}$ with a boundary of the genus zero surface and a unit solid ball $\mathbb {B}^3$. The related algorithm in^29^ was designed to first compute the volumetric harmonic map between $\mathcal {M}$ and $\mathbb {B}^3$ and then, based on the newly discovered variational principle^30^, to compute the OMT map via Brenier’s approach^24^. However, the first step in^29^ for the computation of a harmonic map from $\mathcal {M}$ to $\mathbb {B}^3$ is not satisfied with an OMT map. Very recently, in 2019, Yueh et al.^31^ proposed a novel algorithm for the volume-preserving parameterization from $\mathcal {M}$ to $\mathbb {B}^3$ by minimizing the volumetric stretch energy by modifying the Laplacian matrix with the current volume-stretching factor at each iteration step. However, there are infinitely many volume-preserving maps between $\mathcal {M}$ and $\mathbb {B}^3$. The resulting map by^31^ is, in general, not an OMT map from $\mathcal {M}$ to $\mathbb {B}^3$.

In this work, the applicability of moving a solid (brain or any human organ) from one place to another is investigated. A two-stage OMT (TSOMT) procedure is carried out prior to 3D U-Net training and inference. Step one is to transform the brain into a unit solid ball to ensure the convergence of the OMT. Since implementing a 3D CNN on spherical coordinates is complicated, step two is to transform the unit ball to a cube of size $128 \times 128 \times 128$. Rather than the threefold increase in the number of voxels in another 3D U-Net approach^16^, we decrease the total number of voxels by $76.6\%$. It is our belief that with fewer voxels, 3D U-Net training can easily find a local minimum and thus achieve a better performance. After applying the 3D U-Net to this $128 \times 128 \times 128$ cube, the inverse TSOMT returns the dimension to $240 \times 240 \times 155$. In doing so, without losing much detail, the complexity of the 3D U-Net training is greatly reduced. Task01_BrainTumor, which describes a subset of the data used in the BRATS 2016 and BRATS 2017 challenges, is the Brain Tumor dataset included in the Medical Segmentation Decathlon (MSD) Challenge^32,33^. In this paper, 484 3D brain images from the training set of Task01_BrainTumor are divided into training, validation and testing sets comprising 400, 29 and 55 samples, respectively. Training Dice scores of 0.9781, 0.9637, and 0.9305 and testing Dice scores of 0.9202, 0.8794, and 0.8420 can be achieved at 400 epochs for the whole tumor (WT), tumor core (TC), and enhanced tumor (ET) predictions, respectively.

### Our contributions

The TSOMT procedure maps irregular 3D brain images to a 3D cube with minimal distortion to facilitate the input format of the ML program. The combination of using the TSOMT method and 3D U-Net in the training step, to the best of our knowledge, greatly surpasses previous methods. The idea here is that the transformation preserves the global features of the data, unlike other previous methods. The main contributions of this paper are summarized as follows.

- The properties of our proposed TSOMT and inverse TSOMT methods are thoroughly examined, including their abilities to control the density, preserve the local mass, and minimize both the transportation cost and the conversion loss, when mapping an irregular 3D brain image into a canonical 3D cube. The density can be enlarged by contrast-enhanced histogram equalization to make the various tumors more detectable by the 3D U-Net algorithm. The small standard deviation (SD) in the local mass ratios of the TSOMT for 484 samples in the MSD Challenge shows the robustness of the transport methods.
- An MRI brain image in the MSD Challenge is described by a $240 \times 240 \times 155 \times 4$ tensor. In contrast, the TSOMT method uses only a $128 \times 128 \times 128 \times 4$ tensor to optimally express all the effective information of a brain image, as a large amount of air in the original raw data is completely excluded by the TSOMT deformation. The selection of a $128 \times 128 \times 128$ cube has three advantages: (i) it matches the physical distance among the voxels of the brain image scanned by MRI; (ii) the conversion loss of a 3D brain image between TSOMT and the $128 \times 128 \times 128$ cube is only $0.5\%$, which is satisfactory and appropriate for 3D U-Net training; and (iii) using the U-Net algorithm to target hundreds of training brain images on a workstation equipped with MATLAB in four NVIDIA Tesla V100S 32 GB GPUs satisfactorily considers the limited memory capacity.
- The TSOMT technique differs from the method developed by Isensee et al.^12,16,34^ in that a 3D brain image is covered with 16 randomly selected $128 \times 128 \times 128$ cubes. The overlapping tile strategy^10^ uses several subvoxels as inputs to achieve the seamless segmentation of arbitrarily large images. If each voxel consumes 1 byte, then the size of the input data for one brain image using the method^12,16,34^ would be 32 MB. On the other hand, the TSOMT method transforms each brain image into a $128 \times 128\times 128$ cube that consumes 2 MB, which is more economical in terms of the input size and frees up considerable capacity with which to increase the augmented data using different resources to achieve more accurate and effective results.
- Via the various density settings of the brain image and the rotations of the unit solid ball, each 3D brain image can be used to construct several different augmented tensors. Numerical experiments demonstrate that the Dice scores of the WT, TC, and ET are improved by such augmented data, as shown in Fig. 7. Furthermore, since the TSOMT procedure can skillfully represent the global information of a brain image, we also propose a postprocessing scheme by applying the mirroring and rotation techniques to increase the Dice scores of the WT, TC, and ET. The numerical results demonstrate that this postprocessing scheme can improve the associated Dice scores by one to two percent.
- We implement the TSOMT method and the U-Net algorithm on the MSD Challenge dataset and verify the bias (underfit) and variance (overfit) of the learning algorithm, as shown in Fig. 6. The optimal number of epochs is between 45 and 55. Furthermore, numerical experiments show that after 400 epochs, the training and testing Dice scores reach 0.9781 and 0.9202, respectively, for the WT. The TSOMT and 3D U-Net approaches significantly improve the accuracy of brain tumor detection and segmentation. For each testing case, the TSOMT, U-Net inference, inverse TSOMT, and postprocessing steps can be accomplished in fewer than 200 seconds.

The remainder of this paper is organized as follows. “OMT formulation and preprocessing” details the OMT formulation, including problem statements, numerical algorithms, TSOMT maps and their related properties. The detailed numerical results of the TSOMT are shown in “Convergence verification and conversion loss of the TSOMT map”. “Training setup” shows the U-Net structure and evaluation criterion. The training and testing results are discussed in “Results and discussion”. Finally, “Conclusions” summarizes the results and outlines further research directions.

## OMT formulation and preprocessing

### The OMT problem

We now state the classical OMT problem raised by Monge^22^. Let $(X,\mu )$ and $(Y,\nu )$ be two measurable spaces with probability measures $\mu$ and $\nu$, respectively, having the same total mass $\int _X \,\text {d}\mu = \int _Y \,\text {d}\nu$. A map $T:X\rightarrow Y$ is said to be mass-preserving if $\mu (A)=\nu (T(A))$ and $\mu (T^{-1}(B)) = \nu (B)$ for all measurable sets $A\subseteq X$ and $B\subseteq Y$, respectively. Let $\mathbb {F}_{\mu ,\nu }$ denote the set of all mass-preserving maps from *X* to *Y*. For a given transport cost function $\kappa :X\times Y\rightarrow \mathbb {R}$, the OMT problem with respect to $\kappa$ is to find $T^*\in \mathbb {F}_{\mu ,\nu }$ that minimizes the optimization problem

1 $$\begin{aligned} T^* = \mathop {\mathrm {argmin}}\limits _{T\in \mathbb {F}_{\mu ,\nu }} \int _X \kappa (x, T(x)) \,\text {d}\mu \in \mathbb {F}_{\mu ,\nu }. \end{aligned}$$

### Discrete OMT problem

The original OMT problem (1) raised by Monge does not necessarily contain an optimal solution. Kantorovich relaxed the transport map and first proved the existence and uniqueness of the optimal transport map. However, the relaxed approach of Kantorovich may result in a non-bijective map. In practical applications, the OMT map must be a bijective map. Thus, we should return to the original OMT problem to develop an efficient, reliable and robust numerical method. A discrete version of the OMT problem is described as follows.

Let $(\mathcal {M}, \rho )$ be a 3-manifold with a spherical boundary and a density map $\rho$ on $\mathcal {V}(\mathcal {M})$, where $\mathcal {M}$ is a tetrahedral mesh composed of an ordered vertex set $\mathcal {V}(\mathcal {M})$, an edge set $\mathcal {E}(\mathcal {M})$, a triangular face set $\mathcal {F}(\mathcal {M})$ and a tetrahedron set $\mathcal {T}(\mathcal {M})$. Let $(\mathbb {B}^3, \delta )$ be a unit solid ball with a constant density function $\delta \equiv 1$. We further define the piecewise linear density function of $\rho$ on $\mathcal {T}(\mathcal {M})$ and $\mathcal {F}(\mathcal {M})$ by

2a $$\begin{aligned} \rho (\tau )&= \frac{1}{4} \sum _{i=1}^4 \rho (v_i), ~ v_i\in \mathcal {V}(\tau ), ~ \tau \in \mathcal {T}(\mathcal {M}), \quad \rho (\alpha ) = \frac{1}{3} \sum _{i=1}^3 \rho (v_i), ~ v_i\in \mathcal {V}(\alpha ), ~ \alpha \in \mathcal {F}(\mathcal {M}) \end{aligned}$$

for a given density $\rho (v)$ for $v \in \mathcal {V}(\mathcal {M})$. Furthermore, we define the local mass and local area measure at $v\in \mathcal {V}(\mathcal {M})$ by

2b $$\begin{aligned} m_{\rho }(v)&:= \frac{1}{4} \rho (v) \sum _{v\subset \tau } |\tau |, ~ \tau \in \mathcal {T}(\mathcal {M}), \quad a_{\rho }(v) := \frac{1}{3}\rho (v)\sum _{v\subset \alpha } |\alpha |, ~ \alpha \in \mathcal {F}(\mathcal {M}), \end{aligned}$$

respectively, where $| \tau |$ is the volume of $\tau$ and $| \alpha |$ is the area of $\alpha$. Denote $\mathbf {F}_\rho$ as the set of all mass-preserving simplicial maps $f_{\rho }:\mathcal {M}\rightarrow \mathbb {B}^3$ with respect to $\rho$ satisfying $\rho (\tau )|\tau | = \delta |f_{\rho }(\tau )| = |f_{\rho }(\tau )|$ for each $\tau \in \mathcal {T}(\mathcal {M})$ and $f_{\rho }(\tau ) \in \mathcal {T}(\mathbb {B}^3)$. Note that the linear simplicial bijective map between $\tau$ and $f_{\rho }(\tau )$ is given by the barycentric coordinates on the tetrahedron $\tau$. The discrete OMT problem with respect to the 2-norm $\Vert \cdot \Vert _2$ is to find an $f_{\rho }^*\in \mathbf {F}_\rho$ that solves the optimization problem

3 $$\begin{aligned} f_{\rho }^* = \mathop {\mathrm {argmin}}\limits _{f_{\rho }\in \mathbf {F}_\rho } \sum _{v\in \mathcal {V}(\mathcal {M})} \Vert v-f_{\rho }(v)\Vert _2^2 \, m_{\rho }(v) \equiv \mathop {\mathrm {argmin}}\limits _{f_{\rho }\in \mathbf {F}_\rho } T_c(f_{\rho }), \end{aligned}$$

where $m_{\rho }(v)$ is the local mass at vertex $v\in \mathcal {V}(\mathcal {M})$ as in (2b) and $T_c(f_{\rho })$ is the transport cost of $f_{\rho }$.

It is easily seen that the set $\mathbf {F}_\rho$ is convex from the definition

$$|(\lambda f_1 + (1-\lambda )f_2)(\tau )| := \lambda |f_1(\tau )| + (1-\lambda ) |f_2(\tau )| = \rho (\tau ) |\tau |,$$

where $\lambda \in [0,1]$ for $f_1, f_2\in \mathbf {F}_\rho$ and $\tau \in \mathcal {T}(\mathcal {M})$. The projected gradient method is a natural consideration for solving the discrete OMT problem (3). However, because the size of $\mathcal {V}(\mathcal {M})$ is too large and the projection on $\mathbf {F}_\rho$ requires solving some highly nonlinear functions, we are motivated to propose a further thought. We now introduce the computational method for the discrete OMT problem (3). Without loss of generality, each tetrahedral mesh $\mathcal {M}$ is centralized and normalized so that the center of mass is located at the origin and the volume is $\frac{4}{3}\pi$.

### Numerical OMT algorithm

In this subsection, we first develop an area-measure-preserving method for solving the OMT problem from the boundary $\partial \mathcal {M}$ to a unit ball. Then, using such a ball area-preserving OMT map as an initial boundary map, we develop a homotopy method to compute the desired mass-measure-preserving OMT map.

(i) Discrete OMT problem on $\partial \mathcal {M}$: To minimize the size of the variables in (3), we first consider solving the OMT problem on $\partial \mathcal {M}$. In detail, the area-measure-preserving piecewise linear map $f|_{\partial \mathcal {M}}\equiv g:\partial \mathcal {M}\rightarrow \mathbb {S}^2$ (a unit ball) with $g(v_i) := \mathbf {g}_i \equiv (\mathbf {g}_i^1, \mathbf {g}_i^2, \mathbf {g}_i^3)^\top \in \mathbb {S}^2$, $v_i\in \mathcal {V}(\partial \mathcal {M})$, $i=1, \ldots , n_\mathtt {B}:=\#\mathcal {V}(\partial \mathcal {M})$, for the OMT problem on $\partial \mathcal {M}$ is iteratively used to minimize the area energy functional

4 $$\begin{aligned} \mathcal {E}_A(g) = \sum _{\ell =1}^3 \left\| L_A(g) \, \mathbf {g}^\ell \right\| _2^2 + \lambda \sum _{v_i\in \mathcal {V}(\partial \mathcal {M})} \left\| \mathbf {g}_i- \frac{v_i}{\Vert v_i\Vert _2} \right\| _2^2 a_{\rho }(v_i), \end{aligned}$$

where $\mathbf {g}^\ell = [\mathbf {g}^\ell _1, \ldots , \mathbf {g}^\ell _{n_\mathtt {B}}]^\top$, $\ell =1,2,3$, $a_{\rho }(v_i)$ is defined in (2b), $\lambda$ is a regularization parameter, and $L_A(g)$ is the area-weighted Laplacian matrix, similar to that of the stretch energy minimization algorithm^35^, such that

5 $$\begin{aligned} {[L_A(g)]}_{i,j} = {\left\{ \begin{array}{ll} -\frac{1}{2}\left( \frac{\cot \left( \theta _{i,j}(g) \right) }{\sigma _{g^{-1}}\left( [{v}_i, {v}_j, {v}_k]\right) } + \frac{\cot \left( \theta _{j,i}(g) \right) }{\sigma _{g^{-1}}\left( [{v}_j, {v}_i, {v}_\ell ]\right) }\right) &{}\text{ if } [{v}_i,{v}_j]\in \mathcal {E}(\partial \mathcal {M})\text{, }\\ \sum _{\ell \ne i} -[L_A(g)]_{i,\ell } &{}\text{ if } j = i\text{, }\\ 0 &{}\text{ otherwise } \end{array}\right. } \end{aligned}$$

where $\sigma _{g^{-1}}(\alpha )=\frac{\rho (\alpha )|\alpha |}{|g(\alpha )|}$, which is dependent on $\rho$, is the area-measure stretch factor of *g* on triangular face $\alpha \in \mathcal {F}(\partial \mathcal {M})$ with $\rho (\alpha )$ defined in (2a) and $\theta _{i,j}(g)$ and $\theta _{j,i}(g)$ are two angles opposite to edge $g([v_i, v_j])$.

The first term in (4) is designed to smooth the iterative vector $\mathbf {g}$ to avoid the occurrence of folding so that the resulting map $\mathbf {g}$ is as bijective as possible. The coefficients of $L_A(g)$ in (5) are modified by imposing the area-measure stretch factor $\sigma _{g^{-1}}(\alpha )$ for $\alpha \in \mathcal {F}(\partial \mathcal {M})$ in the denominator. It is expected that the area ratio $\sigma _{g^{-1}}(\alpha )$ between $\rho (\alpha )|\alpha |$ and $|g(\alpha )|$ is close to one for all $\alpha \in \mathcal {F}(\partial \mathcal {M})$ when the iteration (4) converges. The second term in (4) with the regularization parameter $\lambda$ is required to minimize the sum of the distances between $v_i$ and $g(v_i)$ for all $v_i\in \mathcal {V}(\partial \mathcal {M})$.

Algorithm 1 computes the discrete OMT on $\partial \mathcal {M}$. The steps 1–10 of Algorithm 1 provide an initial spherical area-measure-preserving map $\mathbf {g}$, similar to the stretch energy minimization algorithm^31^. To minimize the energy functional (4) and achieve the spherical image constraint, we apply the stereographic projection

6 $$\begin{aligned} \mathbf {h}_i := \Pi _{\mathbb {S}^2}(\mathbf {g}_i) := \frac{\mathbf {g}_{i}^1}{1-\mathbf {g}_{i}^3} + \sqrt{-1} \frac{\mathbf {g}_{i}^2}{1-\mathbf {g}_{i}^3}, ~ i=1,\ldots ,n_\mathtt {B}, \end{aligned}$$

to map the spherical image of $\mathbf {g}$ onto the extended complex plane $\overline{\mathbb {C}}$. The vertices $v_i\in \mathcal {V}(\partial \mathcal {M})$ are also projected by $\mathbf {u}_i := \Pi _{\mathbb {S}^2}(v_i / \Vert v_i\Vert _2)$, $i=1,\ldots ,n_\mathtt {B}$. Then, the energy functional (4) is optimized on $\overline{\mathbb {C}}$ by alternatingly solving $\mathbf {h}$ on the unit disk in association with the southern hemisphere and the inversion of the northern hemisphere in steps 13–18 of Algorithm 1.

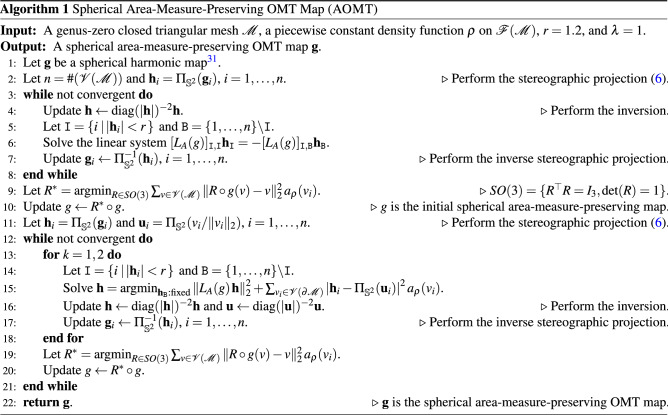



(ii) Homotopy method for the OMT problem on $\mathcal {M}$: We now construct a homotopy $g_t:\partial \mathcal {M}\rightarrow \mathbb {R}^3$ for the boundary map by

$$\begin{aligned} g_t(v) = (1-t)v + t g(v), ~ \text {for } v\in \mathcal {V}(\partial \mathcal {M}), ~t\in [0,1]. \end{aligned}$$

To find the interior map that minimizes the cost (3), we start from the identity map and consecutively update the interior with the boundary map given by $g_t$. Let

7 $$\begin{aligned} {}[0,1]_p = \{0=t_0< t_1< \cdots < t_p = 1\} \end{aligned}$$

be the uniform partition of the interval [0, 1] into *p* subintervals. For $k=1, \ldots , p$, we compute the interior map by solving the linear system

$$\begin{aligned} {}[L_{V}(f^{(k-1)})]_{\mathtt {I},\mathtt {I}} \mathbf {f}^{(k)}_\mathtt {I} = - {}[L_{V}(f^{(k-1)})]_{\mathtt {I},\mathtt {B}} {}[g_{t_k}(v)]_{v\in \mathcal {V}(\partial \mathcal {M})}, \end{aligned}$$

where $f^{(0)}\equiv \text {id}$ and $L_V(f)$ is the mass-weighted Laplacian matrix, similar to the volumetric stretch energy minimization algorithm^31^, such that

8a $$\begin{aligned}{}[L_{V}(f)]_{i,j} = {\left\{ \begin{array}{ll} -w_{i,j}(f), &{} \text {if }[v_i,v_j]\in \mathcal {E}(\mathcal {M})\\ \sum _{\ell \ne i} w_{i,\ell }(f), &{} \text {if }i=j, \\ 0, &{} \text {otherwise} \end{array}\right. } \end{aligned}$$

in which, as in the literature^31,35–37^,

8b $$\begin{aligned} w_{i,j}(f) = \frac{1}{9} \sum _{\begin{array}{c} \tau \in \mathcal {T}(\mathcal {M}) \\ {}[{v}_i, {v}_j] \cup [{v}_k, v_\ell ] \subset \tau \\ {}[v_i, v_j]\cap [v_k,v_\ell ] = \varnothing \end{array}} \frac{|f([v_i,v_k,v_\ell ])| |f([v_j,v_\ell ,v_k])| \cos \theta _{i,j}^{k,\ell }(f)}{\rho (\tau ) |\tau |} \end{aligned}$$

is the modified cotangent weight, where $\theta _{i,j}^{k,\ell }(f)$ is the dihedral angle between $f([v_i,v_k,v_\ell ])$ and $f([v_j,v_\ell ,v_k])$ in tetrahedron $f([v_i, v_j, v_{k}, v_{\ell }])$. Then, the map $f^{(p)}:\mathcal {M}\rightarrow \mathbb {B}^3$ is the desired OMT map $f_{\rho }^*$.

The detailed steps are summarized in Algorithm 2.

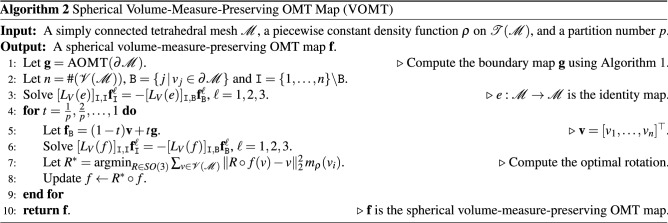



### 3D brain images with a discrete structure

All 3D brain images in the MSD Challenge have $240\times 240\times 155$ voxels, and the irregular 3D brains, on average, account for approximately 16% (between 12% and 20%) of all voxels. However, because of the limitation of the computer’s capacity, the tensor input of the U-Net algorithm^38^ is restricted by $128\times 128\times 128$ voxels, which is undoubtedly a bottleneck for applying the U-Net algorithm to train the 3D brain images for brain tumor segmentation. The most intuitive first thought is to partition the original brain image into several small cubes with $128\times 128\times 128$ voxels or randomly select some small cubes to cover the brain image. In the former, some cubes contain images from the outside of the brain that do not contribute too much to the segmentation training, while in the latter, some cubes have an overlapping area that contains local and partially repeated information, which reduces the segmentation training accuracy. These shortcomings motivated us to introduce the OMT method.

A 3D MRI brain image can be represented as grayscale values in [0, 1] for each grid point (the center of a voxel) of a cuboid $\mathcal {I}$ with rectangular grids $240\times 240\times 155$. If we systematically partition $\mathcal {I}$ into Delaunay tetrahedral meshes with vertices as grid points of $\mathcal {I}$, then a 3D image can further be represented as a piecewise linear map $\varphi :\mathcal {I}\rightarrow [0,1]$ defined by the barycentric coordinates on tetrahedrons. A real 3D brain image can be constructed by a tetrahedral mesh $\mathcal {M}\subseteq \mathcal {I}$ composed of an ordered vertex set $\mathcal {V}(\mathcal {M})$, an edge set $\mathcal {E}(\mathcal {M})$, a triangular face set $\mathcal {F}(\mathcal {M})$ and a tetrahedron set $\mathcal {T}(\mathcal {M})$. Each vertex $v\in \mathcal {M}\subset \mathcal {I}$ can be equipped with a density value

9 $$\begin{aligned} \rho (v) := \exp (\varphi (v)). \end{aligned}$$

As above, the piecewise linear density function $\rho (\tau )$ on $\mathcal {T}(\mathcal {M})$ and the local mass $m_{\rho }(v)$ at $v\in \mathcal {V}(\mathcal {M})$ are given in (2a) and (2b), respectively.

**Figure 1:**
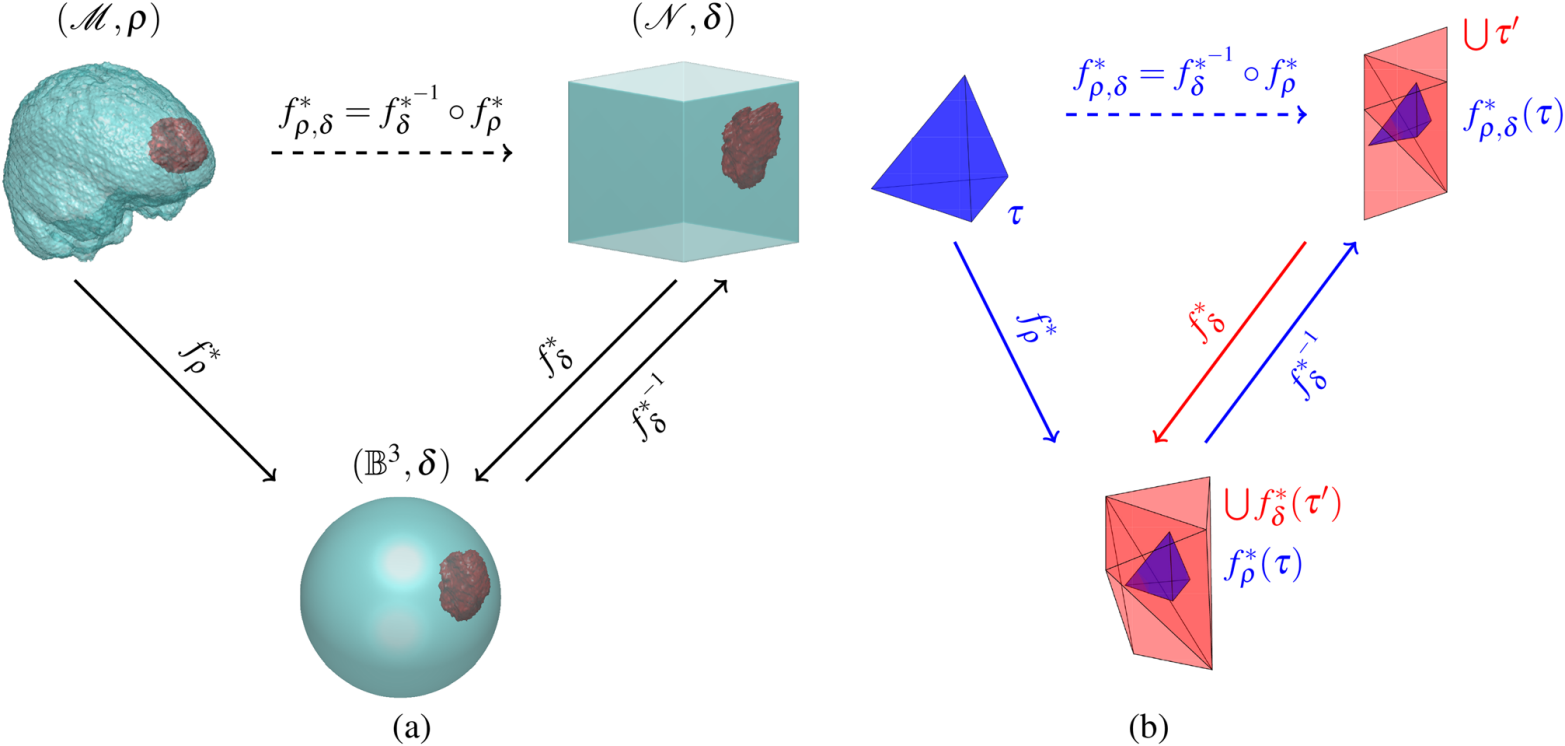
(**a**) A diagram illustrating the construction of the TSOMT map $f^*_{\rho ,\delta }$ between $(\mathcal {M}, \rho )$ and $(\mathcal {N}, \delta \equiv 1)$, where $\mathcal {M}$, $\mathcal {N}$, and $\mathbb {B}^3$ are a 3D brain image, a 3D solid cube, and a 3D unit solid ball, respectively. (**b**) The bijective and linear simplicial map $f_{\rho }^*:\tau \in \mathcal {T}(\mathcal {M})\rightarrow f_{\rho }^*(\tau )\in \mathcal {T}(\mathcal {B}^3)$, $f_{\delta }^*:\tau '\in \mathcal {T}'(\mathcal {N}) \rightarrow f_\delta ^*(\tau ') \in \mathcal {T}'(\mathbb {B}^3)$, and $f_{\rho ,\delta }^*:\tau \in \mathcal {T}(\mathcal {M})\rightarrow f_{\rho ,\delta }^*(\tau )\in \mathcal {T}(\mathcal {N})$.

### Two-stage OMT map

Let $(\mathcal {M}, \rho )$ be a 3D brain image with density function $\rho$ and $(\mathcal {N}, \delta \equiv 1)$ be a solid cube with constant density $\delta \equiv 1$. The canonical 3D cube $\mathcal {N}$ is typically suitable for the tensor input of the U-Net algorithm. However, it is difficult to find an OMT map from $(\mathcal {M},\rho )$ to $(\mathcal {N},\delta )$ directly. The main reason is that cube $\mathcal {N}$ is not in a spherically symmetric domain, so the stereographic projection cannot be used. Therefore, the iteration for minimizing the area energy of (4) exhibits an oscillating behavior and cannot converge. In addition, it is not easy to directly find mass-preserving OMT maps *f* between any two 3-manifolds $(\mathcal {M},\mu )$ and $(\mathcal {N},\nu )$ because the mass stretch factor ($\mu (\tau ) |\tau |) / (\nu (\tau ) |f(\tau )|)$, for $\tau \in \mathcal {T}(\mathcal {M})$, has difficulty converging to one. Hence, we propose a TSOMT map $f^*_{\rho ,\delta } \equiv f^{*^{-1}}_{\delta } \circ f^*_\rho$ (see Fig. 1a) to transform $\mathcal {M}$ into $\mathcal {N}$, where $f_\rho ^*$ is obtained by (3) and $f_\delta ^*$ solves the OMT problem

$$\begin{aligned} f_\delta ^*&= \mathop {\mathrm {argmin}}\limits _{{f}_\delta \in \mathbf {F}_\delta } \sum _{v\in \mathcal {V}(\mathcal {N})} \Vert v-f_\delta (v)\Vert _2^2 \, m_\delta (v) \end{aligned}$$

satisfying $|\tau '| = |f_\delta ^*(\tau ')|$, for all $\tau ' \in \mathcal {T}'(\mathcal {N})$. Here, $\mathcal {T}'(\mathcal {N})$ denotes the Delaunay tetrahedral meshes for cube $\mathcal {N}$. We now show that $f^*_{\rho ,\delta }$ preserves the local mass stretch factor $(\rho (\tau )|\tau |)/|f^*_{\rho ,\delta }(\tau )|$ to be one. In light of Fig. 1b, we have

$$\begin{aligned} \rho (\tau )|\tau |=|f^*_{\rho }(\tau )|&= \sum _{\tau ' \in \mathcal {T}'(\mathcal {N})}^{} |f^*_{\rho }(\tau ) \cap f^*_{\delta }(\tau ')| = \sum _{\tau ' \in \mathcal {T}'(\mathcal {N})}^{} |f^*_{\delta } \circ f^{*^{-1}}_{\delta }\left( f^*_{\rho }(\tau ) \cap f^*_{\delta }(\tau ')\right) |\\&= \sum _{\tau ' \in \mathcal {T}'(\mathcal {N})}^{} |f^*_{\delta }\left( f^*_{\rho ,\delta }(\tau ) \cap \tau ' \right) |~~~~(\text {Since}~ f^*_{\rho ,\delta } = f^{*^{-1}}_{\delta } \circ f^*_\rho )\\&= \sum _{\tau ' \in \mathcal {T}'(\mathcal {N})}^{} |f^*_{\rho ,\delta }(\tau ) \cap \tau '|~~~~~~~~~~~~~~(\text {Since}~ |f^*_{\delta }(\tau ')| = |\tau '|)\\&= |f^*_{\rho ,\delta }(\tau )|, \end{aligned}$$

for $\tau \in \mathcal {T}(\mathcal {M})$, which implies that $f^*_{\rho ,\delta }$ is a mass-preserving map with $\delta =1$.

The main purpose of the OMT problem is to find a mass-preserving map between a 3-manifold with a spherical boundary and a unit solid ball while keeping the deformation between the two manifolds as small as possible. Although we want to seek an OMT map from a 3-manifold to a cube, because the numerical convergence technique does not support this issue, we must first find the OMT map from the 3-manifold to the unit ball. Fortunately, an OMT map from a 3-manifold to a cube can be cleverly obtained by composing the OMT map from the 3-manifold to the unit ball with the inverse OMT map $f^{*^{-1}}_{\delta }$ from the unit ball to the cube, as shown in Fig. 1a.

### Advantages of the TSOMT map

The TSOMT map $f^*_{\rho ,\delta }:\mathcal {M}\rightarrow \mathcal {N}$ is the key issue for tumor segmentation in 3D brain images by using U-Net^38^. It has the following three advantages. (i) The TSOMT technique unifiedly converts each 3D brain image while keeping the minimal deformation and preserving the local mass into a canonical 3D cube uniformly remeshed by $n\times n\times n$. This cube is typically suitable for the tensor input of the U-Net algorithm (see the following subsection for the conversion loss). Furthermore, the mesh grid of the cube can be easily refined or coarsened depending on the accuracy requirement. (ii) The map $f^*_{\rho ,\delta }$ between the 3D cube $\mathcal {N}$ and the brain $\mathcal {M}$, as in Fig. 1, is a one-to-one map. It can skillfully represent the global information of a brain image and provide a more complete density distribution to the supervised learning algorithm, which can train the prediction function to be more accurate. In contrast, the other methods that randomly select several small cubes to cover the brain image^34^ or sliding window combined with the intersection over union technique can express only the local information and reduce the accuracy improvement due to multiple overlapping regions. (iii) The tumor volume with lesions in the brain, in general, accounts for less than one-tenth of the total brain volume. In the supervised learning training data, there is an imbalance between the labeled tumor area and unlabeled healthy area. This phenomenon has a great impact on the intensity of supervised learning. Fortunately, the local measure $\rho (\tau )|\tau | = |f^*_{\rho ,\delta }(\tau )|$ in the TSOMT map is preserved for $\tau \in \mathcal {T}(\mathcal {M})$. This means that the density $\rho (\tau )$ in the tumor is relatively large, and then the local volume in the cube is also relatively large and contains more mesh points; as a result, the tumor and healthy regions in the cube are more balanced, and the disease region can be more accurately determined.

Since the OMT problem is highly nonlinear, a strict mathematical proof is still lacking, but a numerical verification indicates that the computed OMT map is almost one-to-one, and tetrahedral flipping rarely occurs. It can be reasonably asserted that the TSOMT map between the brain image and the cube preserves the local mass and keeps the global information of the density distribution. The resulting cube can easily be uniformly remeshed by $n\times n \times n$ as an input for the U-Net. Based on these advantages, we believe that the TSOMT map provides a more effective tensor input for the U-Net algorithm, which will inevitably obtain more accurate training results than the other existing methods.

## Convergence verification and conversion loss of the TSOMT map

### Verification criterion of convergence

To better understand the convergence behavior of the OMT algorithm, we introduce a global distortion measurement for the accuracy of a mass-preserving map *f* by the total mass-measure distortion defined as

10 $$\begin{aligned} \mathcal {D}_{\mathcal {M}}(f) = \frac{1}{4} \sum _{v\in \mathcal {V}(\mathcal {M})} \left| \frac{\sum _{\tau \supset v}|\tau |\rho (\tau )}{\sum _{\tau \in \mathcal {T}(\mathcal {M})} |\tau |\rho (\tau )} - \frac{\sum _{\tau \supset v}|f(\tau )|}{|f(\mathcal {M})|} \right| . \end{aligned}$$

The smaller $\mathcal {D}_{\mathcal {M}}(f)$ is, the better the mass preservation of *f*. In addition, to express the local distortion of the map *f* computed by the OMT algorithm, we define the local mass-measure stretch ratio $\mathcal {R}_{\mathcal {M}}$ of *f* at a vertex *v* as

11 $$\begin{aligned} \mathcal {R}_{\mathcal {M}}(f,v) =\left( \frac{\sum _{\tau \supset v} |\tau |\rho (\tau )}{\sum _{\tau \in \mathcal {T}(\mathcal {M})} |\tau |\rho (\tau )} \right) / \left( \frac{\sum _{\tau \supset v} |f(\tau )|}{|f(\mathcal {M})|} \right) . \end{aligned}$$

The closer $\mathcal {R}_{\mathcal {M}}(f,v)$ is to one, the better the mass preservation of *f* at *v*.

Furthermore, we verify the bijectivity of the computed OMT map by checking the number of folded tetrahedrons. A map *f* is bijective if the number of folded tetrahedrons in $\mathcal {T}(f(\mathcal {M}))$ is zero. In general, the convex combination maps on 3-manifolds cannot guarantee bijectivity^39^. The ideal way to illustrate the bijectivity is to numerically check the bijectivity rate of the map defined as

12 $$\begin{aligned} \text {Bijective Rate}(f) = \left( 1 - \frac{\#\{\text {folded } f(\tau )\,|\, \tau \in \mathcal {T}(\mathcal {M})\}}{\#\mathcal {T}(\mathcal {M})} \right) \times 100\%. \end{aligned}$$

The method for checking folded tetrahedrons can be found in the literature^31^.

### Four views of the data structures of the training and validation sets

As in a typical medical database, in the MSD Challenge dataset, each brain has four modalities from multiscan views: (i) FLAIR for the WT; (ii) T1 and (iii) T1CE for the ET; and (iv) T2 for the TC.

Let $\{ \mathcal {I}_i \}_{i = 1}^4$ be four cuboids with grids $240 \times 240 \times 155$ denoting the four-view discrete structures of a 3D brain $\mathcal {M}$ composed of a single cuboid $\mathcal {L}$ with grids $240 \times 240 \times 155$ labeled by $\ell (\mathrm{v}) \in \{ 0, 1, 2, 3\}$ on each voxel v in $\mathcal {L}$. The original grayscale value of each grid point (the center of a voxel) of $\{ \mathcal {I}_i \}_{i = 1}^4$ is a nonnegative integer, usually ranging between 0 and 4000. The standard grayscale value of each vertex (the center of voxel) of $\mathcal {I}_i$ can easily be normalized by *(grayscale value - mean)/variance* and a suitable shift into the interval [0, 1]. Let $\varphi _i : \mathcal {I}_i \rightarrow [0, 1]$ be the associated piecewise linear map by the barycentric coordinate and $\{ \mathcal {M}_i\}_{i = 1}^4$ be the brain images of the *i*th view corresponding to FLAIR, T1, T1CE and T2, respectively.

**Figure 2:**
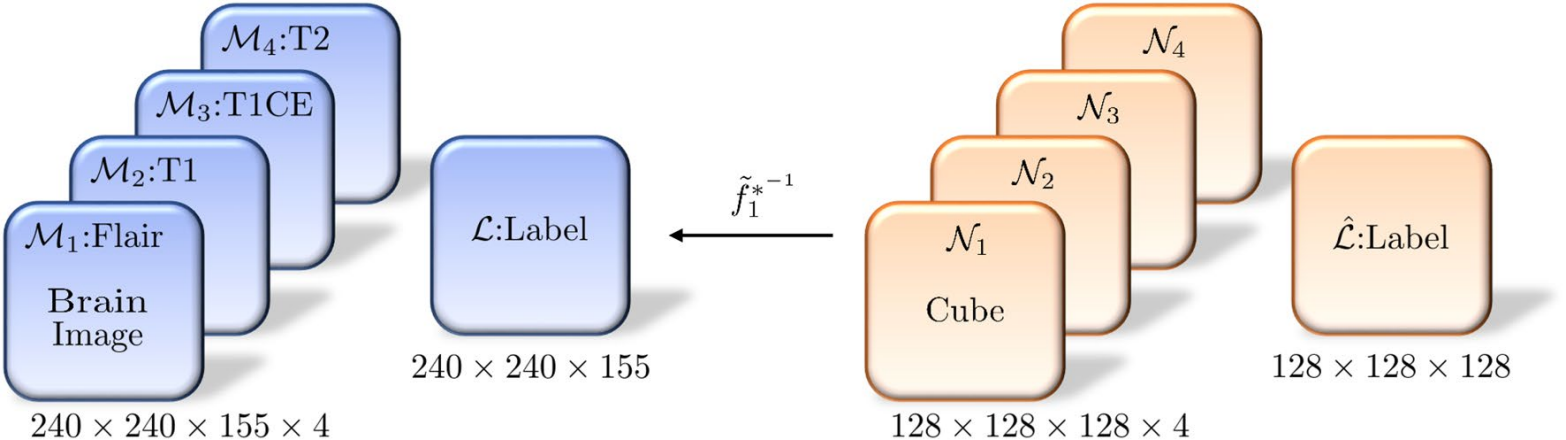
The TSOMT maps between 4-view brains and cubes with $n = 128$.

To clearly distinguish between tumor and nontumor regions in the brain, it is recommended that the standard grayscale of $\mathcal {I}_i$ is enhanced by the histogram equalization algorithm^40^. Since the grayscale of $\mathcal {I}_1$ for FLAIR has a clearer gap between tumors and nontumors, to save computational costs, we enhance the standard grayscale of $\mathcal {I}_1$ by^40^ and obtain the contrast-enhancing grayscale $\tilde{\varphi _1} : \mathcal {I}_1 \rightarrow [0, 1]$. Then, we only construct the OMT map with $\tilde{\varphi _1}$ and share this OMT with four views: FLAIR, T1, T1CE, T2. As in (9) we define $\tilde{\rho _1}(v) := \exp (\tilde{\varphi _1}(v)))$ for each vertex $v\in \mathcal {M}_1$ and construct the TSOMT map

13 $$\begin{aligned} \tilde{f}_1^* \equiv f_{\tilde{\rho _1}, \delta }^* :(\mathcal {M}, \tilde{\rho }_1) \rightarrow (\mathcal {N}, \delta \equiv 1) \end{aligned}$$

with $\mathcal {N}$ being a cube, as in Fig. 1. Let $\{ \mathcal {N}_i\}_{i = 1}^4$ and $\hat{\mathcal {L}}$ be cubes remeshed by the uniform grid points $n \times n \times n$. We consider the following map in Fig. 2 and define the grayscale values and labels on $\mathcal {N}_1 \times \mathcal {N}_2 \times \mathcal {N}_3 \times \mathcal {N}_4 \times \hat{\mathcal {L}}$ by

14 $$\begin{aligned} \left( \varphi _1({\tilde{f}_1^{*^{-1}}}(w)), ~\varphi _2({\tilde{f}_1^{*^{-1}}}(w)), ~\varphi _3({\tilde{f}_1^{*^{-1}}}(w)), ~\varphi _4({\tilde{f}_1^{*^{-1}}}(w)), ~\ell ({\tilde{f}_1^{*^{-1}}}(w)) \right) \in [0, 1]^4 \times \{0, 1, 2, 3\} \end{aligned}$$

where *w* is the center of a voxel in $\{ \mathcal {N}_i\}_{i = 1}^4$ or $\hat{\mathcal {L}}$. Then, $\mathcal {N}_1 \times \mathcal {N}_2 \times \mathcal {N}_3 \times \mathcal {N}_4 \times \hat{\mathcal {L}}$ typically forms effective training input data for the U-Net algorithm based on a set comprising an $n \times n \times n \times 4$ tensor and an $n \times n \times n$ label.

### Numerical convergence of the TSOMT map

**Figure 3:**
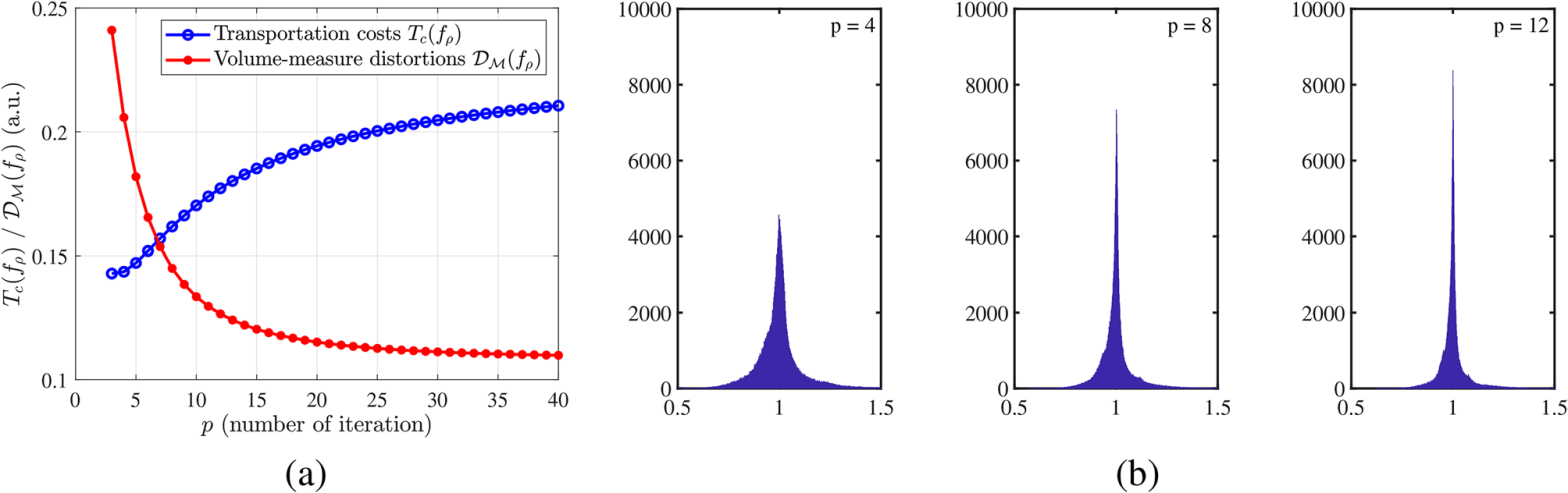
(**a**) The transport costs $T_c(f_{\rho })$ and the total mass-measure distortions $\mathcal {D}_{\mathcal {M}}(f_{\rho })$ vs. the number of partitions *p* of the homotopy method. (**b**) Histogram of the local mass-measure stretch ratio $\mathcal {R}_{\mathcal {M}}(f_{\rho },v)$ when $p = 4, 8$ and 12 for no. 021.

We test 484 3D brain images from the MSD Challenge dataset and compute the OMT maps $f_{\rho }^{*}$ and $f_{\delta }^{*}$ with $n = 128$ and $\rho = \tilde{\rho }_1$ by the numerical OMT algorithm. For a typical 3D brain image, say no. 021, in Fig. 3a, we show the corresponding transport costs $T_c(f_{\rho })$ of (3) in the OMT map $f_{\rho }^{*}$ in blue and the total mass-measure distortions $\mathcal {D}_{\mathcal {M}}(f_{\rho })$ of (10) in red vs. the number of partitions *p* in (7). We see that as *p* increases, the transport cost increases, while the total mass-measure distortion $\mathcal {D}_{\mathcal {M}}(f_{\rho })$ decreases. The reasons are that (i) when *p* increases and becomes sufficiently large, the boundary map of homotopy becomes very fine, which makes the ratio $\max _{\tau \in \mathcal {T}(\mathcal {M})} \rho (\tau )|\tau |/|f_{\rho }(\tau )|$ closer to one and makes $\mathcal {D}_{\mathcal {M}}(f_{\rho })$ undoubtedly smaller; (ii) On the other hand, when *p* decreases, the total mass-measure distortion constraint is more relaxed, which makes the corresponding transport cost smaller. The average of intersections of the blue line and the red line is roughly 8. In Fig. 3b, we plot the histogram of the local mass-measure stretch ratio $\mathcal {R}_{\mathcal {M}}(f_{\rho },v)$ in (11) for $p = 4, 8$ and 12. We observe that the deviations in $\mathcal {R}_{\mathcal {M}}(f_{\rho },v)$ show a downward trend for *p* from 4 to 12. For more detail, in Table 1, we list the transport cost $T_c(f_{\rho }^*)$, the total distortion $\mathcal {D}_{\mathcal {M}}(f_{\rho }^*)$, the mean, the SD, and the number of foldings from $2.2 \times 10^5$ vertices of $\mathcal {M}$ for $p = 4, 8$ and 12. We see that the transport cost and the total distortion for $p = 8$ are between those for $p = 4$ and 12. Therefore, in practice, we reasonably choose $p=8$ for the OMT algorithm.

**Table 1:** No. 021: transport cost $T_c(f_{\rho }^*)$ of (3), total mass-measure distortion $\mathcal {D}_{\mathcal {M}}(f_{\rho }^*)$ of (10), mean, SD and number of folded tetrahedrons for $p = 4, 8$ and 12, respectively.

No. 021	$p = 4$	$p = 8$	$p = 12$
Transport cost $T_c(f_{\rho }^*)$	0.0976	0.1085	0.1157
Total distortion $\mathcal {D}_{\mathcal {M}}(f_{\rho }^*)$	0.0748	0.0430	0.0330
Mean	1.0001	0.9999	1.0002
Standard deviation (SD)	0.1348	0.0842	0.0680
Number of folded tetrahedrons	12	8	6

**Figure 4:**
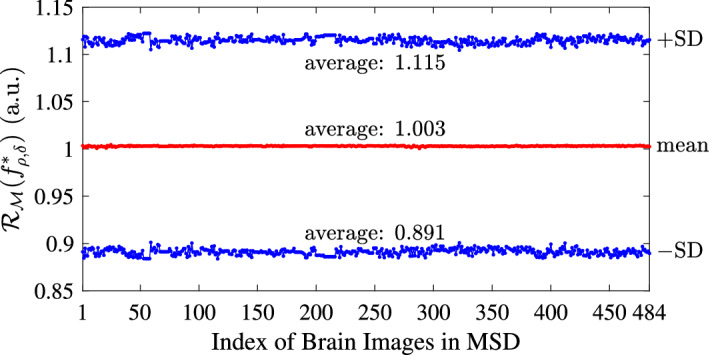
The mean (red) and mean ± SD (blue) of the local mass-measure stretch ratio of the TSOMT maps with $p = 8$ for each of the 484 brain images in the MSD Challenge dataset, as well as the averages of the means and ± SDs over the 484 images.

To show that the map $f_{\rho , \delta }^{*}$ computed by the TSOMT algorithm with $p = 8$ always has satisfactory local distortions, as we expected, in Fig. 4, we show the mean and mean ± SD of the local mass-measure stretching ratio $\mathcal {R}_{\mathcal {M}}(f_{\rho ,\delta }^{*},v)$ for each brain image. Here, $\mathcal {R}_{\mathcal {M}}(f_{\rho ,\delta }^{*},v)$ is defined in (11) with $f = f_{\rho ,\delta }^{*}$. We see that the mean of $\mathcal {R}_{\mathcal {M}}(f_{\rho ,\delta }^{*},v)$ is always extremely close to one, and the SD of $\mathcal {R}_{\mathcal {M}}(f_{\rho ,\delta },v)$ is bounded by 0.112, which is satisfactory.

Furthermore, we verify the bijectivity of the computed TSOMT map by checking the bijectivity rate of the map in (12). It is worth noting that among all 484 maps computed by the TSOMT algorithm, the number of folded tetrahedrons $f_{\rho ,\delta }^{*}(\tau )$ is at most 19, while the number of tetrahedrons of each mesh is at least more than 1 million. As a result, the bijectivity rate of every TSOMT map is larger than $99.99\%$, which is quite satisfactory. Thanks to the large-scale bounded distortion mappings^41^, the folded tetrahedrons can be modified and unfolded into $100\%$ bijective mass-preserving maps by slightly sacrificing the total mass distortion.

### Conversion loss between cubes and original brains

As in the previous section, the TSOMT map transforms irregular 3D brain images into cubes while preserving the local mass and minimizing the deformation, which makes the U-Net algorithm train an effective prediction function for brain tumor segmentation. Since the tensor cube is needed as the input for the U-Net, it is necessary to compare the conversion loss between the original brain images and cubes of various sizes. We first remesh the original brain with roughly $2 \times 10^5$ vertices, which is an appropriate magnitude for the stability of the accuracy.

Once the cuboid $\hat{\mathcal {L}}$ is labeled by $\hat{\ell }(w) \in \{0, 1, 2, 3 \}$ at each voxel center *w* in $\hat{\mathcal {L}}$, we construct the associated conversion label set on a brain image $\mathcal {M} \subseteq \mathcal {L}$ by using the inverse function $\tilde{f}_1^{*^{-1}} \equiv f_{\tilde{\rho }_1, \delta }^{*^{-1}}$ in (13) as follows:

- For a voxel v $\in \mathcal {M} \subseteq \mathcal {L}$, if there is a voxel center $w \in \hat{\mathcal {L}}$ such that $\tilde{f}_1^{*^{-1}}(w) \in$ v, then v is labeled by $\hat{\ell }(w)$;
- Otherwise, v is labeled by $\hat{\ell }(\hat{w})$, where $\hat{w}$ is a voxel center in $\hat{\mathcal {L}}$ and $\tilde{f}_1^{*^{-1}}(\hat{w})$ is the nearest point to the center of v.

Let *A* and *B* denote the label sets of the ground truth and the conversion tumors on $\mathcal {M} \subseteq \mathcal {L}$ for the WT (labeled $\{1, 2, 3\}$), TC (labeled $\{2, 3\}$), and ET (labeled $\{3 \}$), respectively. We define the conversion loss by

15 $$\begin{aligned} 1 - \frac{2|A \cap B|}{|A| + |B|}, \end{aligned}$$

where |*A*| denotes the cardinal number of *A*.

In Table 2, we display the averages of the conversion loss between brains and cubes as in (15) for the WT, TC, and ET of all 484 brain images in the MSD Challenge dataset with typical grid sizes of $96^3$ and $128^3$ by the TSOMT map $f_{\tilde{\rho }_1, \delta }^*$.

**Table 2:** Conversion loss between brains and cubes with grid sizes of $96^3$ and $128^3$, respectively.

TSOMT-$\tilde{\rho }_1$	WT (%)	TC (%)	ET (%)
Conversion loss for $96^3$	1.87	2.06	3.65
Conversion loss for $128^3$	0.51	0.53	0.77

We observe that the deformation of TSOMT-$\tilde{\rho }_1$ with a cube size of $128 \times 128 \times 128$ does not result in a considerable accuracy loss, and the maximum conversion loss for the ET is less than $0.77\%$. On the other hand, although a cube size of $96 \times 96 \times 96$ would conserve considerable computational resources, the maximum conversion loss for the ET is $3.65\%$, which is not conducive to constructing a good prediction function. Therefore, for training an effective prediction function by U-Net, we consciously take the input of the appropriate tensor size of $128 \times 128 \times 128$ for the 3D U-Net algorithm.

Furthermore, in Table 3, we show the average percentages of the WT, TC, and ET in the original brain and in the cube by the TSOMT-$\tilde{\rho }_1$ map with a grid size of $128 \times 128 \times 128$. For instance, the WT accounts for $7.16\%$ of the raw data of the original brain. However, under the enhanced histogram equalization^40^ of the grayscale and TSOMT-$\tilde{\rho }_1$ maps, the WT is enhanced almost twofold in the cube, reaching $13.33\%$. Applying contrast enhancement to the grayscale image^40^ indeed helps to better detect various tumors by the 3D U-Net algorithm.

**Table 3:** The average percentages of the various types of tumors in the raw data with a size of $240 \times 240 \times 155$ and cubes with a size of $128 \times 128 \times 128$ computed by the TSOMT-$\tilde{\rho }_1$ algorithm.

	WT (%)	TC (%)	ET (%)
Tumor in the raw data $(240 \times 240 \times 155)$	7.16	2.65	1.41
Tumor in the cube $(128 \times 128 \times 128)$	13.33	4.88	2.66

## Training setup

### U-Net structure

In this paper, we adopt the U-net algorithm, because medical images have blurred boundaries and complex gradients, more high-resolution information is required. High resolution is used for precise segmentation. The U-net algorithm^10,42^ combines the low-resolution information (providing a basis for object category recognition) and the high-resolution information (providing a basis for precise segmentation and positioning), which is perfectly suitable for medical image segmentation.

To test the effectiveness of the OMT, a typical 3-layer U-Net architecture^10,42^ is used. This network consists of three encoding stages with (128, 64, 32) filters and three decoding stages in reverse order with (32, 64, 128) filters. The main idea is to supplement the decoding stages through a bridge structure with its corresponding encoding stage, which increases the resolution of the output based on this extra information. It has been widely accepted that this bridge structure (encode-bridge-decode) is far better for segmentation applications than a simple encode-decode without bridge. In each encoding stage, there is a 3D CNN followed by batch normalization (BN) and a rectified linear unit (ReLU) activation function, which often does not require dropout for regularization. The same structure is also applied to the decoding and bridge stages. The last convolutional layer was followed by a fully connected layer, and the final layer of the CNN employed a softmax classifier with 2 output nodes for the 2 desired classes. Table 4 shows the total learnable parameters in each layer in the entire network.

**Table 4:** The number of learnable parameters in the each layer of a U-Net model.

Layer	Dimension	Learnable parameters
Encoder-1	128	59,040
Encoder-2	64	332,352
Encoder-3	32	1,328,256
Bridge-1	16	1,770,240
Bridge-2	16	3,540,480
Decoder-1	32	9,177,088
Decoder-2	64	2,294,784
Decoder-3	128	573,952
FC	128	130

A deep learning neural network learns to map a set of inputs to a set of outputs from training data using an optimization process that requires a loss function to calculate the model error. From Table 3, the tumor percentage is small even after OMT and enhances histogram equalization. A well-known deep learning loss function for highly unbalanced segmentations is adopted to ensure convergence^43,44^:

$$\begin{aligned} \text {Loss} = 1 - \frac{2 \sum _{k=1}^K w_k \sum _{m = 1}^M Y_{km}T_{km}}{\sum _{k=1}^K w_k \sum _{m = 1}^M Y_{km}^2 + T_{km}^2}, \end{aligned}$$

where *Y* is the predicted probability, *T* is the ground truth, *K* is the number of classes, *M* is the number of elements along the first two dimensions of *Y*, and $w_k = (\sum _{m=1}^M T_{km})^{-2}$.

The hyperparameters in the U-Nets are chosen as follows: Encoder Depth: 3; Initial Learning Rate: $\alpha _0 = 1.0\times 10^{-4}$; Learning Rate Drop Factor: $F=0.95$; Learning Rate Drop Period: $P = 10$; $L_2$-Regularization: $1.0\times 10^{-4}$; Minimum Batch Size: 8; Epoch: 400.

$$\begin{aligned} \alpha _{\text {Ep}} = \alpha _0 \times (F)^ {\text {Ep} / \text {P}}, ~ \text {Ep}= \text {number of epochs}. \end{aligned}$$

### Data augmentation

Detecting malignant tumors in the brain and precise segmentation are difficult tasks in neurology because it is not easy to find enough well-labeled training datasets. In practice, these training data must be exactly diagnosed by a doctor, properly labeled, and deidentified. Therefore, a well-labeled training set for U-Net for detecting brain lesions is relatively small. Nevertheless, in ML, augmenting the amount of data is an effective way to improve the efficiency of U-Net training. U-Net usually requires multiview images of the same type but different angles for training. For example, the image of a tumor inside the brain is still a tumor after arbitrary rotation or mirroring. According to our experience, the Dice score will increase via simple data augmentations such as rotating, mirroring, shearing and cropping. As in (13), if we additionally construct $\tilde{f}_2^*:(\mathcal {M}, \rho _2) \rightarrow (\mathcal {N}, \delta \equiv 1)$ with $\rho _2 \equiv \exp (\frac{1}{4} \sum _{i=1}^4 \varphi _i))$, where $\varphi _1, \ldots , \varphi _4$ are the piecewise linear maps corresponding to FLAIR, T1, T1CE and T2, then $\tilde{f}_2^*$ forms a mass-preserving TSOMT map from $\mathcal {M}$ to $\mathcal {N}$. Then, by $\{ \tilde{f}_i^* \}_{i = 1}^2$, we perform some data augmentation $\{\mathcal {N}_{1,j} \times \mathcal {N}_{2,j} \times \mathcal {N}_{3,j} \times \mathcal {N}_{4,j} \times \hat{\mathcal {L}_j}\}_{j = 1}^2$, as shown in Fig. 2. This data augmentation can be regarded as a perturbation of the size or shape of the brain tumor. It can effectively form an expanded dataset for our training process.

### Implementation

The 484 3D brain images from the MSD Challenge dataset are divided into a training set, validation set and testing set, where the training set includes 400 samples (indexed from 001-400) and the remaining 29 and 55 samples (indexed from 401-484) comprise the validation and testing sets, respectively.

In practice, we first create a sharp contrast-enhanced grayscale image $\tilde{\varphi }_1: \mathcal {I}_1 \rightarrow [0, 1]$ by using the histogram equalization algorithm^40^, as before, and then compute the TSOMT map $\tilde{f}_1^*$ as in (13), and we share this OMT among the four views, namely, FLAIR, T1, T1CE and T2, to construct four cubes $\{\mathcal {N}_{i,1}\}_{i=1}^4$ and one label set $\hat{\mathcal {L}}_1$, as in (14). Similarly, by using the TSOMT map $\tilde{f}_2^*$, we can construct $\{\mathcal {N}_{i,2}\}_{i=1}^4$ and $\hat{\mathcal {L}}_2$. After all the preprocessing is complete, 400 brain samples are expanded into 800 cube samples. Next, we use the U-Net algorithm to train three nets for the WT $\{1,2,3\}$, TC $\{2,3\}$ and ET $\{3\}$:

- Net 1 for the WT: $\{0\}, \{1,2,3\}$.
- Net 2 for the TC: $\{0,1\}, \{2,3\}$.
- Net 3 for the ET: $\{0,1,2\}, \{3\}$.

After Net 1, Net 2, and Net 3 are trained, we use the following definition to predict the tumor labels of the brains in the training set and testing set.

Our calculations are implemented in MATLAB R2020a. The training is carried out in a Tesla V100S PCIe 32 GB$\times 4$ workstation and is stopped at 400 epochs^45^. Each epoch takes approximately 12 minutes. The computational time of the OMT is 20 seconds per iteration on a personal computer equipped with an NVIDIA GeForce RTX 2080 GPU. Thus, the inference including TSOMT preprocessing, U-Net prediction, and inverse TSOMT processing takes 200 seconds to complete on the same PC.

### Flowchart of the segmentation maps

**Figure 5:**
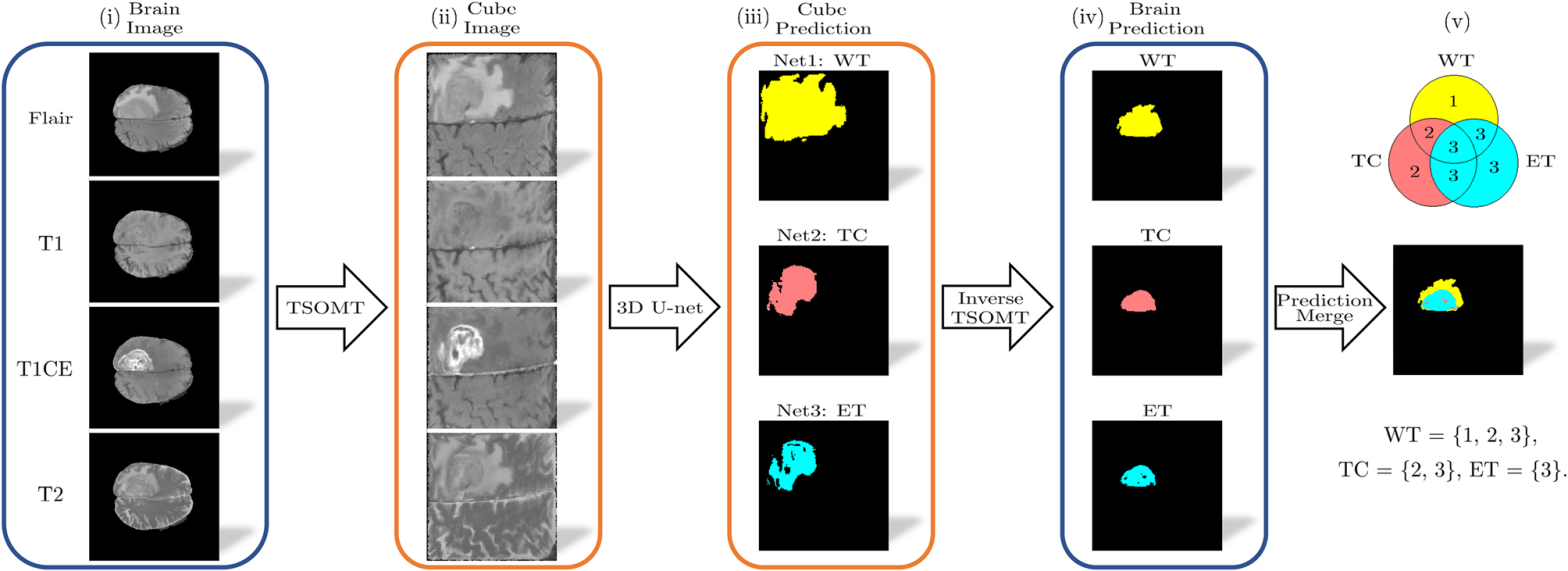
Flowchart of the segmentation maps: (i) input the brain image, (ii) utilize the TSOMT method to transform the brains into cubes, (iii) predict the WT (yellow), TC (red), and ET (blue) through 3D U-Nets Net 1, Net 2, and Net 3, respectively, (iv) reverse the cube prediction back to a brain prediction via the inverse TSOMT method, and (v) merge the WT, TC, and ET.

To further visualize the flowchart for 2D cross-section images, Fig. 5 illustrates the whole inference process. Four brain images (FLAIR, T1, T1CE, and T2) were first transformed to each cube by the TSOMT separately. The original brain size of $240 \times 240 \times 155 \times 4$ is reduced to $128 \times 128 \times 128 \times 4$ cube images with 4 modalities. The cube images are processed by Net 1, Net 2, and Net 3 to obtain three segmentation images with a size of $128 \times 128 \times 128$. The inverse TSOMT procedure then converts the segmentation images to $3\times (240 \times 240 \times 155)$. These three images can then be merged with simple logic to obtain the final predicted brain results ($240 \times 240 \times 155$). By matching the ground truth with the prediction voxel by voxel, the accuracy is then calculated as a ratio of false predictions to the entire tumor.

### Evaluation criteria

In this paper, we use the Dice score, sensitivity (recall), specificity, precision and 95th percentile of the Hausdorff distance (HD95) as indicators to evaluate the segmentation performance of our proposed algorithm. Consider the confusion matrix as

**Table:** 

	Ground truth (yes)	Ground truth (no)
Prediction (yes)	TP	FP
Prediction (no)	FN	TN

where TP is the number of true positives, TN is the number of true negatives, FN is the number of false negatives, and FP is the number of false positives. The indicators are defined as follows:

**Table:** 

Dice score	Sensitivity	Specificity
$\frac{2\text {TP}}{2\text {TP} + \text {FP} + \text {FN}}$	$\frac{\text {TP}}{\text {TP} + \text {FN}}$	$\frac{\text {TN}}{\text {TN}+\text {FP}}$

For a fixed brain tumor, as in (15), let *A* and *B* denote the positive ground truths and predictions, respectively, for the WT, TC, and ET. Then, the HD is defined as

$$\text {HD} = \max \{\max _{a\in \partial A}\min _{b\in \partial B}\Vert a-b\Vert _2, ~\max _{b\in \partial B}\min _{a \in \partial A}\Vert a-b\Vert _2\}.$$

HD95 is similar to the HD, and it calculates the 95th percentile of the distance between the boundary points in *A* and *B*. In the following, we consider applying the OMT technique on the MSD Challenge dataset to obtain a good training set and make highly accurate predictions.

## Results and discussion

### Dice score and loss plot

**Figure 6:**
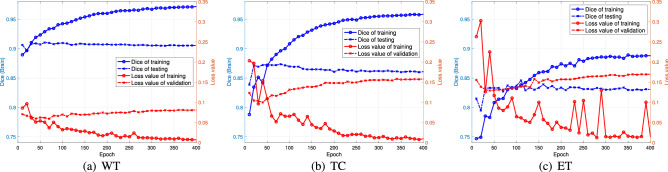
The Dice scores for training and testing (blue lines) and the loss functions for training and validation (red lines) vs. the number of epochs for the WT, TC, and ET.

A deep learning neural network learns to map a set of inputs to a set of outputs from training and validation data using an optimization process that requires loss functions for training and validation sets to calculate the model error. For convenience, we implement the TSOMT procedure with a size of $96\times 96\times 96$ and the U-net algorithm with a minibatch size of 8 on the MSD Challenge dataset with 400, 29 and 55 samples for training, validation and testing, respectively. Figure 6 plots the loss values of the training set (red solid line) and the validation set (red dotted line), as well as the Dice scores of the training set (blue solid line) and the testing set (blue dotted line) for the WT, TC and ET every 10 epochs. The two red curves corresponding to the loss functions in each subfigure of Fig. 6 form a variance (overfit) when the number of epochs nears 400 and a bias (underfit) when the number of epochs is near 10; the optimal number of epochs for the WT, TC and ET is between 45 and 55. The two blue curves of the Dice scores in each subfigure of Fig. 6 intersect at epoch 50 for the WT and TC and at epoch 100 for the ET with Dice scores of 0.9072, 0.8608 and 0.8305, respectively, which coincides with the optimal number of epochs for the bias and variance.

The plots of the blue solid lines for training in Fig. 6 can usefully reflect the training of the model. We see that the Dice scores for the WT, TC, and ET do not increase significantly after 400 epochs and approach 0.9716, 0.9318 and 0.8879, respectively. The trends of both the Dice scores and the loss values show a typical training history. These results are also consistent with the findings reported in Table 2, where the conversion loss increases in the order of ET < TC < WT, and in Table 3, where the tumor percentages decrease in the order of WT > TC > ET. A small tumor percentage implies fewer balanced data. Thus, the Dice score is smaller.

However, the Dice scores for the WT, TC, and ET in the testing set remain constant throughout the whole optimization process, where the training set often does not reflect the characteristics of the testing set. This drawback can be improved if more brain tumor cases are included in the training set. By reducing the size of each brain tumor case via the TSOMT method, our approach can take more training cases if needed.

Here, the 484 brain images of the MSD Challenge dataset are divided into a training set and a testing set with 400 and 84 samples, respectively. Table 5 lists the numerical results by Net 1, Net 2 and Net 3 at epoch 400 for the WT, TC, and ET. The conversion losses between the $128 \times 128 \times 128$ cubes and brains for the WT, TC, and ET are quite reasonable (within $0.4\% \sim 1.3\%$). With U-Net training, the conversion losses are compatible with those in Table 2, where U-Net training is not included. Additionally, the Dice scores for the brains and cubes show a decreasing trend of WT > TC > ET because of the small tumor percentage.

**Table 5:** The Dice scores of the WT, TC, and ET in the cubes and brains in the training and testing sets with 400 epochs.

Epochs	Dice score (cubes)			Dice score (brains)		
400	WT	TC	ET	WT	TC	ET
Training	0.9822	0.9686	0.9447	0.9781	0.9637	0.9305
Testing	0.9105	0.8557	0.8369	0.9065	0.8502	0.8302

Other measures of the voxelwise overlap in the segmented regions such as the sensitivity, specificity, and HD95 evaluate the distances between segmentation boundaries and are all calculated as shown in Table 6. For all those matrices, it is expected that the training results should be better than the testing results. However, HD95 for the ET is the opposite (10.5229 for the training set vs. 6.011 for the testing set). There were 12 cases without ET labels in the 400-sample training set and zero cases in the 84-sample testing set. Those 12 cases dramatically increase the HD. The HD values without those 12 cases are enclosed in parentheses.

**Table 6:** Sensitivity, specificity and HD95 for the WT, TC, and ET for the training and testing sets with 400 epochs.

Epochs	Sensitivity			Specificity			HD95		
400	WT	TC	ET	WT	TC	ET	WT	TC	ET
Training	0.9861	0.9743	0.9420	0.9998	0.9999	0.9999	1.3673	1.6673	10.5229 (1.231)
Testing	0.9229	0.8771	0.8099	0.9989	0.9995	0.9996	9.5475	9.7921	6.0110

### Postprocessing

It is known that simple postprocessing can considerably enhance the segmentation performance by providing additional information^4^. Since the OMT and 3D U-Net have produced significant training improvements, our main focus is to use postprocessing for testing. To provide additional information, mirroring and rotation techniques are added during the testing process. Since the TSOMT map can skillfully represent the global information of a brain image and provide a complete density distribution to the U-Net algorithm, we apply the mirroring and rotation techniques during testing to increase the Dice scores of the WT, TC, and ET. Let $\mathcal {C}_1 \times \mathcal {C}_2 \times \mathcal {C}_3 \times \mathcal {C}_4$ denote the cube corresponding to the TSOMT map of the testing data $\mathcal {N}_{1} \times \mathcal {N}_{2} \times \mathcal {N}_{3} \times \mathcal {N}_{4}$. Here, we add four schemes $\{ \text { R}_k \}_{k=1}^4$ to the testing cube $\mathcal {C}_1 \times \mathcal {C}_2 \times \mathcal {C}_3 \times \mathcal {C}_4$, where $\text { R}_1$ represents a 90 degree counterclockwise rotation, $\text { R}_2$ indicates mirroring from left to right, $\text { R}_3$ denotes mirroring upside down, $\text { R}_4$ represents mirroring left to right, followed by a 90 degree counterclockwise rotation, provided by the MATLAB functions rot90, fliplr and flipud. For each scheme $\text { R}_k$, we perform the following two steps: (i) rotation or mirroring $\mathcal {C}_1 \times \mathcal {C}_2 \times \mathcal {C}_3 \times \mathcal {C}_4$ by $\text { R}_k$ to obtain a new cube $\tilde{\mathcal {C}}_1 \times \tilde{\mathcal {C}}_2 \times \tilde{\mathcal {C}}_3 \times \tilde{\mathcal {C}}_4$ and (ii) predicting the probability $p_k$ of voxel $\tilde{\mathcal {C}}_1 \times \tilde{\mathcal {C}}_2 \times \tilde{\mathcal {C}}_3 \times \tilde{\mathcal {C}}_4$ being a tumor. Let $p_0$ be the predicted probability of voxel $\mathcal {C}_1 \times \mathcal {C}_2 \times \mathcal {C}_3 \times \mathcal {C}_4$ being a tumor. Then, we use the following rule to determine the final judgment of this voxel: If $(\sum _{k=0}^4 p_k) > (1 - \sum _{k=0}^4 p_k)$, then this voxel is a tumor; otherwise, it is healthy.

Table 7 shows the brain Dice scores of the WT, TC, and ET for the schemes $\{ \text { R}_k \}_{k=1}^4$ at epoch 400 and the percentage improvement. The numerical results demonstrate that the proposed schemes $\{ \text { R}_k \}_{k=1}^4$ can improve the associated Dice score ranging from one to two percent. The whole testing procedure is as follows: TSOMT, U-Net inference, inverse TSOMT, and mirroring and rotation.

**Table 7:** The Dice scores of the WT, TC, and ET in brains with postprocessing and the corresponding improvement percentages.

Epochs	Dice score (brain)			Improvement		
400	WT	TC	ET	WT	TC	ET
Testing	0.9202	0.8794	0.8420	$1.37\%$	$2.92\%$	$1.18\%$

### Effect of augmented rotations

**Figure 7:**
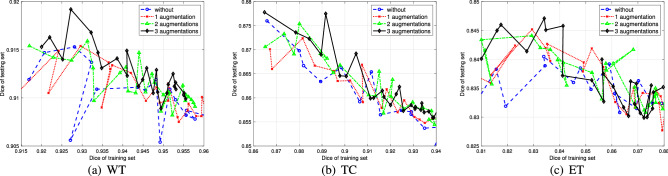
The Dice scores of the WT, TC, and ET in the training and testing sets vs. the number of augmentations.

As indicated in the third item of the main contributions, the TSOMT method can substantially increase the augmented data to achieve more accurate and effective results. We apply the U-Net algorithm with 250 epochs to the MSD Challenge dataset (484 samples) preprocessed by the TSOMT method. To increase the augmented data, we construct densities $\rho _i, i = 2, 3, 4$ and randomly rotate the 400 unit solid balls with density $\rho _i$ in the first-stage OMT for $i = 2, 3, 4$. Figure 7 plots the Dice scores of the training set vs. the testing set from 50 to 250 epochs by the blue, red, green and black lines for the training data without augmented rotations and the training data with one, two and three augmented rotations, respectively. We see that the training data with more augmented rotations have a certain effect on improving the Dice score of the testing data. This result coincides with the advantage that we introduce the TSOMT procedure to represent an effective 3D brain image by a $128 \times 128 \times 128$ cube.

### 3D prediction

**Figure 8:**
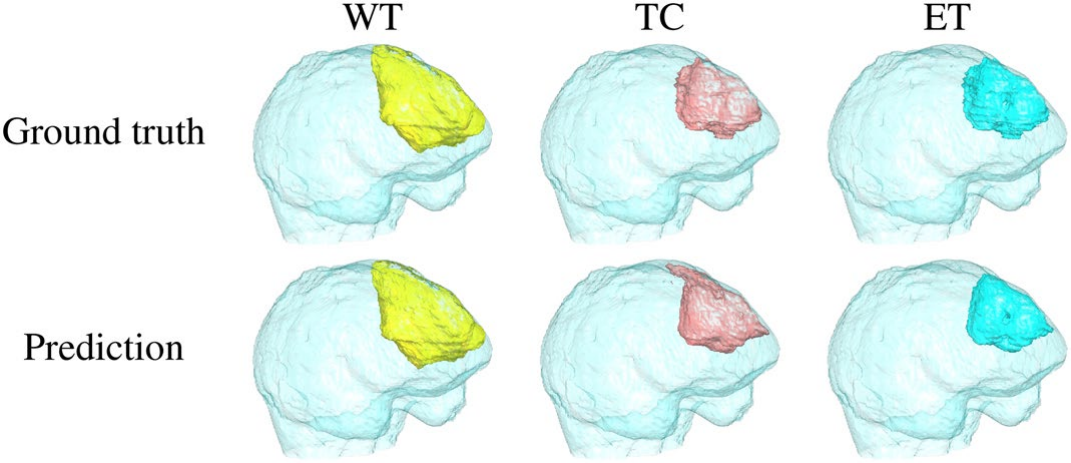
The ground truths and predictions for the WT (yellow), TC (red) and ET (blue) of case no. 410 with the best WT case (Dice score = 0.9738).

To evaluate the performance of the proposed segmentation algorithm, we compare the segmentation output with the ground-truth labels, which are prepared by field experts. The qualitative 3D segmentation results vs. the ground-truth labels are plotted in Figure 8 for case 410, where the brain Dice scores of the WT, TC, and ET are found to be 0.9738, 0.9188, and 0.8585, respectively. In addition to one case, for the training set (nos. 001-400), the best, average and worst cases for the WT are 0.9948, 0.9852 and 0.9619, respectively. This finding implies that the segmentation results of the lesion areas achieved by the proposed model are very close to the ground-truth labels. Our method (TSOMT+U-Net+inverse TSOMT) significantly outperforms other state-of-the-art approaches. For the testing set (nos. 401-484), the best, average and worst cases for the WT are 0.9738, 0.9193 and 0.7312, respectively. Again, our method (TSOMT+U-Net+inverse TSOMT+postprocessing) shows good results. All 484 predicted NII files are included online at https://reurl.cc/9ZZ12v.

### Worst testing cases

**Figure 9:**
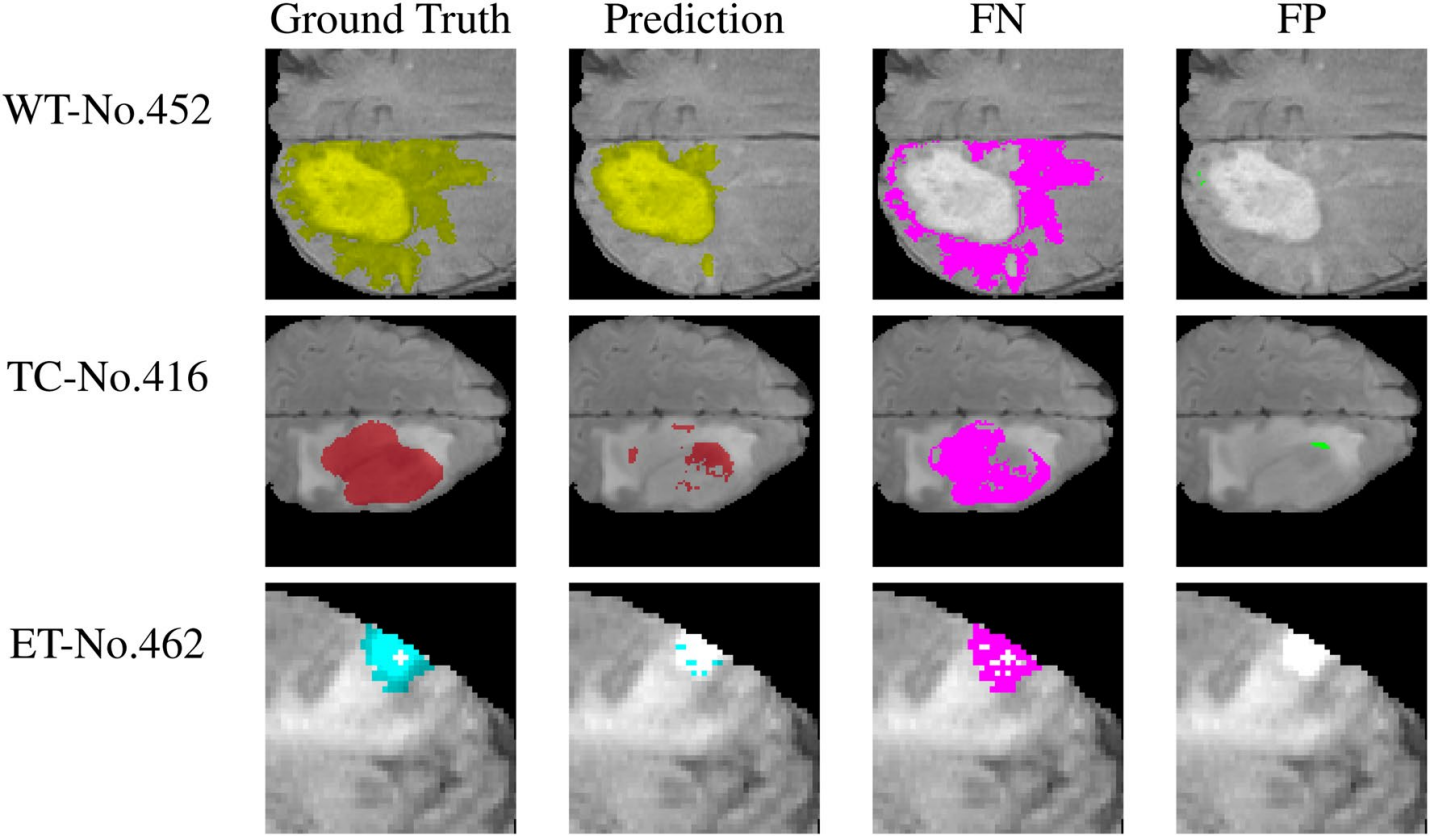
The worst WT (yellow) and TC (red) and ET (blue) testing cases: Cross-sections of no. 452 at 95 along the z-axis, no. 416 at 123 along the z-axis, and no. 462 at 56 along the z-axis for the ground truths, prediction, false negative (purple) and false positive (green).

The worst testing cases are further investigated. Among them, three cases (408, 430, and 452) had WT Dice scores below 80%, three cases (417, 453, and 459) had TC Dice scores below 70%, and three cases (427, 459, and 462) had ET Dice scores below 60%. Figure 9 plots cross-sections of the ground truth and predictions for the cases with the most incorrectly predicted voxels (452, 426, and 462). The incorrect predictions are further grouped into false negatives and false positives in the figure. The false negative:false positive voxel ratios for the WT, TC, and ET in all testing cases are found to be 1.128, 1.529, and 1.904, respectively. Our approach shows false negatives rather than false positives, and the trend of WT < TC < ET conceptually agrees with previous observations. Furthermore, the TSOMT and inverse TSOMT methods do not alter the deficiency qualitatively because the conversion losses from cubes to brains for these three cases are all smaller than 1.2%. The conversion loss is too small to induce any new failure mode. In addition, the brain Dice scores of (452, 416, 462) for the WT, TC, and ET were found to be (0.7312, 0.9549, 0.9239), (0.9260, 0.6371, 0.7435), and (0.8348, 0.7280, 0.4130), respectively. False predictions of the WT, TC, and ET are seemingly independent from each other. This finding justifies the use of three U-Nets.

A typical incorrect WT prediction is shown for case no. 452. Based on this typical type of brain tumor, we observe that the false negative and false positive regions for the best testing case that are incorrectly distinguished almost always occur at the junction boundaries of different levels of tumors and nontumors. This deficiency can hopefully be reinforced by boundary image processing^46^, boundary segmentation^47^, and edge detection^48,49^ methods. On the other hand, for the worst testing case (no. 452) with a more severely spreading tumor, there is a relatively large region on the outermost periphery of the tumor that is very vague and difficult to distinguish, regardless of whether it is positive or negative. We see that the tumor type of this case significantly reduces the prediction accuracy. Inaccurately predicted tumor locations also occur on the interfaces of different labels of tumors or nontumors, but they seem to expand outward more. This is indeed a challenging problem of how to improve image segmentation techniques.

TC and ET false predictions are more like an embedded type than a boundary type in the WT. Case no. 416 (TC) and case no. 462 (ET) are plotted. In these two cross-sections, false positives are negligible. The TC and ET cases are inherently less accurate because of the smaller regions (see Table 7 for the testing case results). Additionally, the prediction has many scattered necrotic regions in the TC and ET. As a result, the false negative exhibits a Swiss cheese-like structure and greatly reduces the prediction accuracy. This deficiency, different from that of the WT, can hopefully be reinforced by contour filtering and processing techniques^50^ during postprocessing.

### Testing on the BRATS 2020 dataset

In the above sections, we train a U-Net model for 400 3D brain images from the MSD Challenge dataset with augmented rotations by using the TSOMT method, the U-Net algorithm, and the postprocessing correction technique and successfully achieve satisfactory Dice scores for the 84 MSD testing samples, as shown in Table 7.

We now use the well-trained U-Net model to infer brain tumors for the testing brain images from the BRATS 2020 dataset^1–3^. We first check the 369 brain images of the training data in BRATS 2020 and find that 210 samples are duplicates of samples in the MSD dataset. We then take the remaining 159 samples as the testing data and infer brain tumors. The Dice scores of the WT, TC, and ET are 0.9002, 0.7387 and 0.7723, respectively, which appear satisfactory. All 159 predicted NII files are included online at https://reurl.cc/2rrrQE.

Furthermore, an online evaluation platform for BRATS 2020 was recently opened and provides 125 unlabeled brain images for testing. The feedback Dice scores of the WT, TC and ET predicted by our U-Net model are 0.8829, 0.7289 and 0.7297, respectively (see the online record of the team “GIMILab” at https://www.cbica.upenn.edu/BraTS20/lboardValidation.html). The resulting scores are not too high but are still acceptable. The main reason could be that the current testing data have various formats or flaws that have not been standardized. We hypothesize that this testing dataset needs to be classified, which constitutes our research topic in the next phase. We believe that the TSOMT technique developed in this paper has numerous advantages and good efficiency.

## Conclusions

This work mainly introduces the TSOMT technique to the research area of 3D medical image segmentation. We first propose an efficient and reliable numerical algorithm for the computation of the OMT map. Then, we use it to develop the TSOMT for transforming an irregular 3D brain image into a cube while maintaining minimal deformation. The TSOMT map is one-to-one and mass-preserving and minimizes the transport costs.

The concept of representing an irregular brain image with a unit cube with minimal deformation was first proposed in the research field, which is particularly beneficial to the U-Net algorithm’s input format for creating training and testing sets. Expressing brain images as cubes greatly reduces the training input set size from $240 \times 240 \times 155$ to $128 \times 128 \times 128$ and reduces the computational time required for training. In addition, this preprocessing mean by the TSOMT map does not cause a considerable loss of accuracy. In contrast, the conversion loss under $128^3$ grid points on the cube that we set is usually between only $0.51\%$ and $0.77\%$. The transport cost, distortion, and conversion loss are all carefully examined. The small SD in the local mass ratios of TSOMT for 484 cases shows the robustness of the transform.

Another advantage of the OMT technique is that it can first perform histogram equalizations on the density of the original brain tumors. Generally, the tumor site with higher density accounts for less volume in the brain image. Nevertheless, we can utilize the merit of the mass-preserving property of the TSOMT to increase the proportion of brain tumors in the cube. This technology is very conducive to the effectiveness of U-Net learning for tumor segmentation.

The training accuracy at 400 epochs reached 0.9822 for the WT on the cube using a 3D U-Net. The inverse TSOMT method returned the dataset to the original size of $240 \times 240 \times 155$ and lowered the accuracy to 0.9781 for the WT on the brain during the conversion loss. This TSOMT, U-Net inference and inverse TSOMT approach significantly improves the brain tumor detection and segmentation accuracy. The training data with more augmented rotations have a certain effect on improving the Dice score of the testing data. For the testing cases, a simple mirroring and rotation postprocessing technique with an accuracy improvement of one to two percent is added to the whole flow. For each new case, the overall flow can be completed in fewer than 200 seconds.

The high accuracy in our numerical experiments can once again reflect the superiority of the TSOMT. The associated cube holds almost all the global information of the brain image and is suitable for the U-Net input. Intuitively, the TSOMT combined with U-Net can master the essential learning rules and has a good chance of realizing a high-precision system for brain tumor segmentation. Therefore, the TSOMT map can be said to be superior to the other preprocessing methods. Thus, expanding the dataset with different tumor types in combination with the virtue of the TSOMT conversion can indeed have expected potential in the field of 3D medical image segmentation.
